# Global migration: Implications for international business scholarship

**DOI:** 10.1057/s41267-022-00565-z

**Published:** 2022-11-14

**Authors:** Aida Hajro, Chris Brewster, Washika Haak-Saheem, Michael J. Morley

**Affiliations:** 1 Leeds University Business School, University of Leeds, Leeds, LS2 9JT UK; 2 Vienna University of Economics and Business, 1020 Vienna, Austria; 3 Henley Business School, University of Reading, Reading, RG6 6UA UK; 4 Dubai Business School, University of Dubai, P.O. Box 14143, Dubai, United Arab Emirates; 5 Kemmy Business School, University of Limerick, Limerick, V94 T9PX Ireland

**Keywords:** global migration, international business scholarship, multinational enterprises, cross-border activities of firms

## Abstract

Migration is increasingly viewed as a high-priority policy issue among politicians, intergovernmental organizations, NGOs, and civil society throughout the world. Its implications for the private sector, for economic prosperity, and for the cross-border activities of firms are undeniable and likely to grow in importance. Yet, despite its relevance to International Business, treatment of migration in the mainstream International Business literature has been limited. In this contribution, we set out key aspects of migration that are germane to International Business. Specifically, we suggest recent migratory shifts are transforming important elements of the context in which multinational enterprises operate, with significant implications for their international human resource management practices, for firms’ entry modes and market selection approaches, and for the manner in which international strategies are formulated and implemented. We offer a research agenda to motivate International Business scholars to study global migration in more depth and to reevaluate the generalizability of aspects of their theories in light of developments in global migration.

## Résumé

La migration est de plus en plus considérée comme une question politique hautement prioritaire par les politiciens, les organisations intergouvernementales, les ONGs et la société civile du monde entier. Ses implications pour le secteur privé, pour la prospérité économique et pour les activités transfrontalières des entreprises sont indéniables et vont probablement prendre de l'importance. Pourtant, malgré son importance pour les affaires internationales, la littérature dominante de celles-ci n’accorde encore que peu d’attention à l’étude de la migration. Dans cette contribution, nous abordons les aspects clés de la migration qui comptent pour les affaires internationales. Plus spécifiquement, nous suggérons que les récents changements migratoires transforment des éléments importants du contexte dans lequel les entreprises multinationales opèrent, et ce avec des implications significatives pour leurs pratiques de gestion des ressources humaines au niveau international, pour leurs approches de sélection des marchés et de modes d'entrée, ainsi que pour la manière dont leurs stratégies internationales sont construites et mises en œuvre. Nous proposons un programme de recherche pour inciter les chercheurs en affaires internationales à étudier de manière plus approfondie la migration mondiale et à réévaluer la généralisabilité de certains aspects de leurs théories à la lumière de l'évolution de la migration mondiale.

## Resumen

La migración se considera cada vez más una cuestión política prioritaria entre los políticos, las organizaciones intergubernamentales, las ONG y la sociedad civil de todo el mundo. Sus implicaciones para el sector privado, para la prosperidad económica y para las actividades transfronterizas de las empresas son innegables y es probable que su importancia aumente. No obstante, a pesar de su importancia para Negocios Internacionales, el tratamiento de la migración en la literatura principal de Negocios Internacionales ha sido limitado. En esta contribución, exponemos los aspectos clave de la migración que son relevantes para Negocios Internacionales. Específicamente, sugerimos que los recientes cambios migratorios están transformando elementos importantes del contexto en el que operan las empresas multinacionales, con implicaciones significativas para sus prácticas de gestión de recursos humanos internacionales, para los modos de entrada de las empresas y los enfoques de selección de mercados, y para la forma en que se formulan e implementan las estrategias internacionales. Ofrecemos una agenda de investigación para motivar a los estudiosos de Negocios Internacionales a estudiar la migración global en mayor profundidad y a reevaluar la generalizabilidad de aspectos de sus teorías a la luz de los desarrollos en la migración global.

## Resumo

Migração é cada vez mais vista como uma questão política de alta prioridade entre políticos, organizações intergovernamentais, ONGs e a sociedade civil em todo o mundo. Suas implicações para o setor privado, para prosperidade econômica e para as atividades transfronteiriças das empresas são inegáveis e provavelmente crescerão em importância. Porém, apesar de sua relevância para Negócios Internacionais, o tratamento da migração na literatura dominante de Negócios Internacionais tem sido limitado. Nesta contribuição, apresentamos aspectos chave da migração que são pertinentes a Negócios Internacionais. Especificamente, sugerimos que recentes mudanças migratórias estão transformando relevantes elementos do contexto no qual empresas multinacionais operam, com significativas implicações para suas práticas internacionais de gestão de recursos humanos, para modos de entrada de empresas e abordagens de seleção de mercado, e para a maneira como as estratégias internacionais são formuladas e implementadas. Oferecemos uma agenda de pesquisa para motivar acadêmicos de Negócios Internacionais a estudar migração global com mais profundidade e reavaliar se aspectos de suas teorias são generalizáveis dados os desenvolvimentos na migração global.

## Abstract (zh)

移民越来越被世界各地的政治家、政府间组织、非政府组织和民间社会视为一个高度优先的政策问题。它对私营行业、经济繁荣和企业跨境活动的启示是不可否认的， 且可能会变得越来越重要。然而，尽管它与国际商务相关， 主流的国际商务文献对移民的研究是有限的。在这篇文章中， 我们列出了与国际商务密切相关的移民问题的关键面。具体而言， 我们认为最近的迁移转变正在改变跨国企业经营环境的重要因素， 对其国际人力资源管理实践、公司的进入模式和市场选择方法以及国际战略的制定与实施方式产生重大影响。我们提供了一个研究议程， 以激励国际商务学者更深入地研究全球移民， 并根据全球移民的发展重新评估其理论的各个方面的通用性。

## Introduction

One of the most discussed phenomenon of our time has been the rise in global migration (de Haas, Castles, & Miller, 2020; 2017). Some 281 million people now reside in a country different from the place of their usual residence; of these, approximately two-thirds are labor migrants (IOM, 2021). Against the backdrop of this development, migration may be considered a “frontier phenomenon” (Verbeke, Von Glinow & Luo, 2017), necessitating new lines of inquiry through a business lens (Hajro, Caprar, Zikic, & Stahl, 2021). Indeed, some scholars have even proposed a redirection of International Business (IB) research toward the study of migration as one of the pressing “grand challenges” in global business (Buckley, Doh, & Benischke, 2017).^1^

Behind the raw numbers are important nuances and subtleties that feature less prominently in the discourse on migration (Hajro, Zilinskaite, & Baldassari 2022a; King, 2012). The migrant total remains a small percentage of the world’s total population (3.6%), implying that most people stay in their countries of birth (IOM, 2021). In addition, various changes have occurred in migration trends and patterns, three of which are of relevance to our arguments here. First, the number of migrants has increased since 2000, with the result that the international migrant proportion of the world population has risen by nearly a full percentage point in the past two decades (IOM, 2021). Second, there have been two major transformations in global migration patterns, namely changes in the geography and composition of global migrants. Compared with 75 years ago, today’s migrants hail from a wider array of countries and settle in an ever-smaller number of countries (de Haas et al., 2019). In addition, the evidence suggests that the average educational level among migrants has increased over the years (Czaika, 2018).

Set against this backdrop, we examine the implications of changes in global migration for multinational enterprises (MNEs), and highlight how migration-related topics could usefully be incorporated into the IB research domain. Our contribution is threefold. First, we call attention to the relevance and significance of global migration for IB scholarship by delineating the characteristics (alterations in patterns) and implications of migration for the contemporary context in which MNEs operate. Among the features we identify here are the multiplicity of countries of origin and the increased educational levels of immigrants in developed economies, the fluctuations in labor supply in emerging market economies, and the economic, political, and sociocultural impacts of migration on migrants’ sending and receiving countries. Second, we identify three key ways in which global migration affects existing IB research, namely: the changing nature of location and firm-specific advantages in light of access to human capital; the implications of migration for firms’ entry modes and market selection approaches; and how migration impacts firms’ organizational culture and approach to strategy. Third, we suggest a route forward for IB research on important questions at the intersection of global migration, IB, and society, questions of particular relevance to policy and practice, as well as specifically to MNEs.

The insights we offer are informed by both the literature in IB, and the literature in other social science disciplines that have a much longer engagement with the topic of migration: sociology, anthropology, population geography, political science, and economics. As Verbeke et al., (2017: 2) observed, in the spirit of embracing different sources of “knowing” about a phenomenon, a pluralistic approach encompassing the borrowing of ideas from other fields can serve to “strengthen the predictive and explanatory capacity of extant theoretical frameworks” in our field.^2^ To be clear, the objective of our paper is not to offer a wide-ranging literature review of IB and migration, nor a detailed treatise on their intellectual progression over time. Rather, we suggest that, while global migration is a distinct phenomenon with an “own identity” (Buckley et al., 2017: 1053), an understanding of its contours can assist in advancing aspects of IB research on MNEs.

Our analysis is presented under three main themes as follows: (1) changing patterns of global migration; (2) transformation of the international business context; and (3) implications of global migration for the activities, strategies, and decision-making processes of MNEs.

## Changing patterns of global migration

We use the International Organization for Migration (IOM) and the United Nations Department of Economic and Social Affairs definition of an international migrant as someone who moves away from their country of usual residence across an international border, temporarily or permanently, and for whatever reason. Most of the descriptive statistics we use rely on this definition, which combines all the different categories of very diverse migrants that have relationships to businesses. Such relationships are vested in the fact that firms benefit not only from migrants’ labor and skills but also from the innovations that they produce, the networks that they leverage, and the consumption in which they engage (de Haas et al., 2020; Hajro, Zilinskaitė, Gibson, Baldassari, Mayrhofer, Brewster, & Brannen, 2022b).

To depict the changes in global migration patterns, for three reasons we selected the post-World War II period as our starting point. First, the period following World War II saw the increasing reversal of earlier colonization engaged in by various European states, a development which brought with it important consequences for immigration to Europe’s traditional settler societies (de Haas et al., 2019). Second, the development of the field of IB can be traced to the post-World War II period: Hymer’s (1960) seminal contribution was among the first serious efforts to explain foreign direct investment at the firm level. Third, although we rely on different sources, e.g., IOM and the International Labor Organization, to describe changes in the patterns of global migration, we obtained valuable insights from the Determinants of International Migration (DEMIG) project and the Global Bilateral Migration Database (GBMD). The flow of bilateral migration for 34 reporting countries, and from 236 countries since the end of World War II, can be found in the DEMIG country-to-country database. The GBMD contains bilateral migration population data for 226 countries from 1960 to 2000 (Özden, Parsons, Schiff, & Walmsley, 2011). Taken together, the GBMD and DEMIG databases permit analyses and insights into the ebb and flow of migration patterns that were previously largely undetectable (Czaika & de Haas, 2014). Given the different databases we have to draw upon, we are, on occasion, required to look at different time periods, but the overall focus on the changes that have occurred since the end of World War II remains.

### Changes in Geography of Global Migration

The most significant post-World War II changes in geography of global migration comprise: (1) declining emigration from Europe coupled with increasing immigration to Europe, (2) increasing emigration from Asia and Latin America, (3) the rise of new global migration magnets, and (4) many developing countries entering a migration transition (Czaika & de Haas, 2014; de Haas et al., 2019). We elaborate upon these developments in more detail below.

First, following post-World War II decolonization, European migration patterns shifted significantly from emigration to immigration (Czaika & de Haas, 2014). Another particularly noteworthy alteration occurred after 1989 and the fall of the Berlin Wall. Countries in Central and Eastern Europe became important sources of labor for Western Europe, creating a significant migration from east to west. Migration patterns from Africa to Europe also altered, with increased numbers of migrants from sub-Saharan Africa joining those from the Maghreb, which had been the dominant source of migrants from Africa to Europe (Flahaux & de Haas, 2016). Immigration to Europe from the Middle East, Latin America, and Asia also increased in the twentieth century, the combination of which remade the mix of migrants’ origins across Europe (de Haas et al., 2019).

Second, as fewer Europeans were emigrating, immigrants from Asia were heading for countries traditionally known as targets for immigration, most notably the USA, Canada, Australia, and New Zealand, marking another shift in migration patterns. Thus, for example, it is estimated that of all international migrants who moved to another region of the world, some 75% were Europeans, but, by 2017, the proportion of migrants who originated from Europe had shrunk to 22%. Concomitantly, the percentage of Asian migrants engaging in long-distance migration has significantly increased (de Haas et al., 2020). The estimated 8% of Asian emigrants who moved outside Asia in 1960 increased to 58% in 2017 (de Haas et al., 2019). The Indian population of the USA in 1960 swelled to become today the second largest Asian immigrant group, surpassed only by the Chinese (Hanna & Batalova, 2020; Zong & Batalova, 2017). The result is that these countries of origin have lost considerable talent, with the OECD suggesting that by 2010 approximately 3.5 million Chinese and Indian college graduates worked in advanced economies. These same two sending countries accounted for 28% of migrating inventors during the 2000s (Kerr, 2019). Similarly, a decline in the global wealth ranking of Latin America resulted in the region’s huge drop in immigration, particularly the waning in immigration from Europe, which had been the traditional origin of immigrants in the Americas (Czaika & de Haas, 2014), and accelerated emigration to traditional European countries of settlement.

A third change lies in the attractiveness of the Arab Gulf countries and the bustling economies of countries such as Singapore, Korea, and Japan, all serving as alternatives to North America, Europe, Australia, and New Zealand (IOM, 2021). In the context of the Gulf countries, the oil shock resulted in them becoming attractive destinations for global migration, originally for workforces from oil-poor Arab countries, such as Sudan, Egypt, and Jordan, but gradually also for countries in Asia such as Indonesia, India, the Philippines, Pakistan, and Nepal (Fargues, 2011; Shah, 2013). Together with this, the economic growth in East Asia, starting in Japan and then in Singapore, Taiwan, South Korea, and Hong Kong, resulted in redirected patterns of migration focused on these expanding economies (Skeldon, 2006).

Finally, with respect to the geography of migration, a fourth shift exists in the transitioning of countries such as Yemen, the Philippines, Turkey, Morocco, and Egypt from immigration countries into emigration countries, because of varying levels of development, educational advances, growing infrastructure, and stronger integration into the international system (Czaika & de Haas, 2014; IOM, 2020).

### Changes in the Composition of Global Migration

The trend of an increasingly diverse cohort of migrants headed for a shrinking number of destinations manifested itself in the changes in net immigration and net emigration countries between 1960 and 2000. Net immigration countries declined to 78 from 102, while net emigration countries went up from 124 to 148 (de Haas et al., 2019). Presently, about two-thirds of international migrants reside in one of just 20 countries (IOM, 2022).

An overall increase in worldwide educational levels is reflected in the characteristics of migrants who increasingly tend to fill needs for skilled labor in advanced economies (Czaika, 2018). Countries, including for example, Canada, Australia, New Zealand, and the UK, have shifted in recent years toward systems based on points, arguing that such an approach confers greater flexibility in targeting and securing specific skills. Such programs attempt to balance demands and job openings with labor availability. Several EU countries have followed the example of the early adoptees of such programs (Platonova, Schuster, Desiderio, Urso, & Bürkin, 2013).

However, the existence of these programs has not yielded a level playing field in attracting high-skilled immigrants. Some 70% of the qualified migrants to the OECD went to the USA, the UK, Canada, and Australia. The USA historically has attracted almost half the OECD countries’ qualified migrants and about a third of the total worldwide. The volume of skilled migration to these four countries, coupled with growing policy efforts to attract qualified migrants to European countries, implies that the global race for talent will remain stiff yet unequal, with implications for location advantages of nations (Kerr, Kerr, Özden, & Parsons, 2017). To compete for skilled labor, countries must be able to offer the scale and diversity needed to drive innovation, with many countries still working at not only attracting qualified people from all over the world but also capitalizing on their talents. China, as a case in point, highlights that size or economic progress are not necessarily the answer, as immigrants there are less than 0.1% of the population and its reputation for openness is poor (Kerr, 2019).

The changes in skill levels of global migrants are consistent with globalization trends and processes over the past several decades, in which immigration policies have increasingly favored immigrants with better educational qualifications and higher skills, while barriers to entry to those without such credentials are retained (de Haas et al., 2019).

## Transformation of the international business context

We have identified three important implications of changes in global migration patterns for the contemporary context in which MNEs operate. These implications cover the multiplicity of countries of origin and increased educational levels of immigrants in developed economies, fluctuations in the unqualified labor supply in emerging market economies, and the continuing effects of economic, political, and sociocultural transformations in migrants’ sending and receiving countries***.***

### Multiplicity of Countries of Origin and Increased Educational Levels of Immigrants in Developed Economies

Changes in the geography and the composition of global migration have altered aspects of the contemporary context in which MNEs operate. After World War II, migration consisted mostly of large numbers of semi-skilled or low-skilled people moving internationally through relatively well-regulated channels to supply labor for advanced economies, such as people from the former Yugoslavia to Austria or from Mexico to the USA. Today, those routes have multiplied in terms of origins and destinations (de Haas et al., 2019; Vertovec, 2019). In contrast to the large immigrant groups during the period 1950-1970, today “newer, smaller, transient, more socially stratified, less organized, and more legally differentiated immigrant groups comprise global migration flows” (Vertovec, 2010: 86).

Furthermore, many of these individuals are university-educated and concentrated in cosmopolitan areas (IOM, 2018). As an illustration, the top 100 metropolitan areas in the USA account for 85% of first-generation immigrants counted in the 2010 US Census, and, of those areas, New York, Chicago, Miami, Los Angeles, and San Francisco accounted for over 40% of the US foreign-born population (Singer, 2013). These are also the cities where the headquarters of many non-US MNEs are based. The growth in these highly skilled, diverse categories of migrants has transformed the workforce composition of many MNEs, with, as we will argue in the next section, important implications for global staffing policies and diversity practices.

### Fluctuations in Unqualified Labor Supply in Emerging Market Economies

There has been considerable debate about the notion of “brain drain”, of developed countries “pulling” expensively trained, highly qualified, and much-needed workers from developing countries (Li, McHale, & Zhou, 2017; Tung, 2008). However, as the COVID-19 pandemic highlighted, unqualified workers are also needed to make society work (Caligiuri, De Cieri, Minbaeva, Verbeke, & Zimmermann, 2020). Their emigration, coupled with the changing structure of labor markets, has also created a shortage of workers in some of the “sending” economies, such as Malaysia and Hungary (Inotai, 2019). In some instances, collaborations have been forged between intergovernmental and non-profit organizations, policymakers, and MNEs to develop ways forward in the labor market for unqualified migrant workers (Jureidini, 2016), although, as has been highlighted by Hajro et al. (2021), IB scholars have had limited input into these discussions.

### Economic, Political, and Sociocultural Impacts in Migrants’ Sending and Receiving Countries

Altered patterns of migration, coupled with new means of communication and transportation, have transformed the way individuals and institutions are linked across national borders (Vertovec, 1999), and given rise to a growing cohort of so-called “trans-migrants,” who are former migrants now fully at home in their new countries, but also linked in numerous ways to their old countries (Schiller, Basch, & Blanc, 1995). The new transnational linkages that have been forged have changed how former migrants perceive themselves in their destination countries, and have yielded significant changes in individual orientations, consumption patterns, and processes of economic development in both their host and originating countries (Castles, 2010). As highlighted by Portes (2003: 877–878), “the combination of a cadre of regular transnational activists with the occasional activities of other migrants adds up to a social process of significant economic and social impact for communities and even nations. While from an individual perspective, the act of sending a remittance, buying a house in the migrants’ hometown, or traveling there on occasion have purely personal consequences, in the aggregate they can modify the fortunes and the culture of these towns and even of the countries of which they are part.”

Thus, migrant transnational ties can alter the value systems and norms of people across different countries and regions (Kyle, 2000; Vertovec, 2004). Overseas migrant communities not only modify aspects of their countries of origin through their “back home” remittances and broader sociomaterial transfers but also participate in shaping and modifying the rules, policies, and institutions of their destination countries through their interactions with locals. Evidence suggests that their founding of organizations to serve their own cultural, economic, and even legal needs over time may create an environment designed to emulate aspects of the one they left behind (Shukla & Cantwell, 2018). The ties with the countries they left are most regularly maintained by the most established and better-educated immigrants. The probability of transnational entrepreneurship increases by 1% for every year of education; a high school diploma can equal a 173% increase in transnational activity (Portes, 2003). If migrants continue their worldwide trend of gaining more education (Czaika, 2018), the future is likely to bring more activities across national boundaries and deeper links between sending and destination countries.

Overall, the worldwide proliferation of migrant transnational ties is a phenomenon of considerable significance and one that deserves greater attention in the IB literature, because it has important implications for the contemporary context in which MNEs operate.

## Implications of global migration for the activities, strategies and decision-making processes of MNEs

We classify the implications of global migration for MNEs into three main categories: (1) the changing nature of location and firm-specific advantages in light of access to human capital; (2) the implications of migration for firms’ entry modes and market selection approaches; and (3) the impact of human capital flows on firms’ organizational culture, and approach to strategy.

In this section, we also provide suggestions on how to extend existing IB theoretical perspectives by incorporating different insights from migration studies. Migration research is a multidisciplinary field that dates back to Ravenstein (1885). An in-depth overview of existing theoretical perspectives in migration research is beyond the scope of this paper. Nevertheless, to inform and orient readers, we first provide a broad summary of the main migration theories and subsequently elaborate upon the interrelationship between migration and the multinational firm.

Migration research is generally considered to be informed by two main paradigms, namely ‘functionalist’ and ‘historical–structural’ perspectives. Adopting a functionalist perspective largely treats migration as a positive development which contributes to greater equality between and within societies (de Haas, 2021). Both push–pull models and neoclassical migration theories are seen to arise from this functionalist perspective. Push–pull models focus on the identification of factors which are likely to serve to push people out of locations of origin and pull them into new destinations (Lee, 1966). Neoclassical theory views migration as a phenomenon that occurs as a result of geographical differences in demand and supply of labor. In this way, it plays a central role in optimizing the allocation of production factors (Harris & Todaro, 1970). Conversely, scholars adopting an historical–structuralist perspective consider migration as one of a range of indicators of the unequal terms of trade between developing and developed countries (Massey et al., 1998). Viewed in this way, the very act of migration further contributes to the uneven development between countries whereby the resources of poor countries are further exploited in order to make the rich ones richer (Castles & Kosack, 1974).

While both the ‘functionalist’ and ‘historical–structural’ paradigms offer explanatory power in providing a certain understanding of the nature of migration, they have been critiqued on the basis that they do not treat the issue of human agency and its role in migration (de Haas et al., 2020). As a result, interest in this question of the agency that migrants possess, coupled with the range of ways in which they actively and creatively overcome structural constraints, has grown since the 1980s. Here, perspectives advancing our understanding of ‘networking’ and ‘transnationalism’ have proven especially important. Migration network theory focuses on the role of social capital (de Haas, 2021). In particular, it describes how migrants build and sustain social ties, both with other migrants in the host locations and with people in the country of origin (Massey et al., 1993). Transnational theory emphasizes the role of evolving identity formation and transition arising from ongoing transnational activities engaged in by migrant communities. Among those particular activities of interest in this regard are economic ones, such as the sending of remittances or the setting up of entrepreneurial ventures “back home”, but also cultural, political, and religious activities, in which migrants engage (Portes, 1999).

Overall, what is apparent from the key theoretical perspectives that have been employed heretofore is that migratory movements have been predominantly explicated through the interacting of both macrostructures (e.g., efforts by the states to control migration) and microprocesses (e.g., particular values, practices, and ties of the migrants themselves). However, what has not been addressed is the relationship between migration and the firm (de Haas, 2021; Hajro et al., 2022b), and it is to this that we now turn our attention.

### The Changing Nature of Location and Firm-Specific Advantages in Access to Human Capital

An underlying assumption in IB research has been that location assets are location-bound (Dunning, 2000; Narula & Santangelo, 2009), and in principle available to all firms in that location (Mudambi, Narula, & Santangelo, 2018). Human capital is one such asset. Depending on its availability, MNEs may prefer to locate economic activity in one country or location rather than another (Graf & Mudambi, 2005; Kedia & Mukherjee, 2009). However, changes in migration patterns have altered the balance of location-specific human capital advantages with, in many developed countries, an internationally mobile, highly diverse labor pool that is ever more accessible. At the same time, MNEs in some emerging  market economies report a limited availability of unqualified labor, posing serious impediments to their production (Inotai, 2019; Jureidini, 2016). Such labor shortages can push up wages in these countries, perhaps causing foreign investors to look elsewhere (Grieveson, 2019). The changes in the availability of both qualified and unqualified migrants have a range of implications for MNEs.

#### Qualified migrants

In many developed countries, businesses face labor market shortages and are lobbying governments to allow them to bring in qualified foreign employees. Large global firms regularly make the case that foreign employees they want to hire are essential for the growth of the firm and its future. In this context, companies play a crucial role in the selection and employment of migrants. Yet, their actions and involvement have not been widely recognized in the multidisciplinary literature on migration (Kerr, Kerr, & Lincoln, 2015). Likewise, most discussions on global staffing in IB have overlooked important codependences and interactions between migrant employees, MNEs, and governments in favor of studies of assigned expatriates (McNulty & Brewster, 2019). Despite the recent COVID-19 pandemic, these expatriate assignments are unlikely to disappear, but MNEs’ reliance on these employees may decrease (Collings & Isichei, 2018; Ravasi, Salamin, & Davoine, 2015), with potentially important implications for coordination, control, knowledge transfer, and global leadership development within the MNEs (Caligiuri et al., 2020). A recent consultancy study of 350 MNEs in more than 25 countries found ongoing shifts in the types of assignments, with traditional expatriate posts declining in favor of short-term assignments or available posts being filled by migrants (KPMG, 2020). Several factors lie at the heart of these changes, including supply side issues (dual careers aspirations, limited number of women in international management, and repatriation concerns), expatriate failure, and the growing emphasis on qualified migrants (Collings & Isichei, 2018). The result is that MNEs can and do increasingly make use of migrants to fill key positions in both subsidiary operations and at headquarters (Hajro et al., 2022a).

This has important implications for the conventional (assigned expatriates) mobility policies and practices of MNEs. First, migrants are characterized by diversity (including ethnic, cultural, linguistic, and religious diversity), have pursued different migration channels resulting in variations in their legal status, and have different educational attainments (Vertovec, 2010). This diversity creates different experiences, with many migrants experiencing a high degree of intersectionality. Fitzsimmons, Baggs, & Brannen, (2020) demonstrated that characteristics such as race, mother tongue, and gender influence both pay and attainment of supervisory positions among migrants, with the international orientation of employing firms moderating this impact. However, there is much to discover, for example, about how qualified migrants with intersecting identities contend with the seemingly contradictory experiences of being needed but not wanted, and how organizations can support them in their integration efforts. In particular, a deeper understanding of three overlapping contexts in which qualified migrants are embedded, i.e., intersectional (characterized by different stigmas ascribed to migrants’ intersecting identities), organizational (characterized by MNEs’ policies and practices), and societal (characterized by host-country entry requirements and standards of equal treatment for migrants), could prove especially illuminating.

Second, MNEs often fail to fully capitalize on the human resource opportunities represented by migrants as an important reservoir of global talent (Kerr, 2019). Misgivings about the quality of foreign academic and professional qualifications often result in immigrants failing to secure jobs for which they are otherwise qualified (Hajro et al., 2022a). Consequently, they end up being underemployed. Their status as migrants and the dynamics of the labor market often confine them to lower status occupations that work against their overall well-being and their opportunities for advancement (Hajro et al., 2021), as well as representing an underused resource for the employer. To give an example, in 2011 in the EU, almost 45% of qualified migrants from third-country nations were in jobs that did not reflect their educational levels, with approximately 12% being “highly overqualified” for the work they were performing (IOM, 2012). Support programs that HRM departments commonly use to aid expatriates may not be sufficient to overcome these barriers.

Third, the pathway to migration in many countries reflects a complex interplay among firms, government, and universities, with the latter serving as a “doorway” for students from foreign countries to enter the workforce of destination countries (Kerr, 2019). The interface between these elements offers considerable potential for future research. With increasing reliance on virtual technologies, most recently during the COVID-19 pandemic, and the rise in online educational programs, students may become free to live anywhere in the world. What implications does this have for firm-specific advantages of developed country MNEs?

Fourth, reflecting the mobility of well-educated people and the demand for their talents, qualified immigrants are often a larger share of the talent that accumulates in developed countries such as the USA, an accumulation that tends to build on itself (Kerr, 2019). To a large extent, national immigration policies shape the mobility of such talent and the direction of its flow. The interplay between MNEs and governments influences this international flow, but how this relates to the economic and locational benefits that destination countries derive from it is largely unexplored. In other words, it is unclear how the actions of MNEs affect international migration flows, or how these flows in turn link to the economic advancement and location advantages of migrant-receiving countries. This has implications for the politics of immigration generally (Kerr, 2019), and for whether MNEs take a political role and become part of the solution to some of the challenges that arise (Scherer & Palazzo, 2011).

#### Unqualified migrants

Businesses are also encountering increasing labor supply gaps among unqualified workers. Here, their lobbying of national governments to allow migrants to fill these gaps has often been less successful. The points-based systems meant to encourage qualified immigrants act against unqualified ones. Governments frequently set policies that restrict the use of unqualified foreign workers, a stance exemplified by the Malaysian government’s “foreign workers first out” policy after 2008 (Jordaan, Oostendorp, & Kinuthia, 2012). It has been argued that, faced with these paradoxical tensions and the serious challenges they present, IB scholars have acted like “semi-interested bystanders” (McGahan, 2019: 111), so that we do not know what implications the changes in country-specific advantages of unqualified labor have for the activities of MNEs. Do MNEs with production operations in these countries choose to relocate their facilities to regions with sufficient labor supply? Do they increase their investment in automation to compensate? Or do they engage in efforts to lobby governments to loosen immigration restrictions?

To facilitate recruitment of migrant workers, many firms rely on private recruitment agencies (Zilinskaite & Hajro, 2020). Unqualified migrant workers in sectors such as construction, agriculture, and services are prone to abuse by such agencies, leaving them vulnerable (Özçelik, Haak-Saheem, Brewster, & McNulty, 2019). In extreme cases, deception about the conditions and nature of work can lead to detrimental contract substitution and human trafficking for labor exploitation (Jureidini, 2016), yet we know little about the policies and operations of these agencies or how MNEs interact with them. Furthermore, while responses to the pandemic may give rise to the reshoring of production (Brakman, Garretsen, & van Witteloostuijn, 2021), what happens to low-status jobs *per se* remains unclear. Will there be an inescapable need to increase the number of migrant workers? And, if yes, what implications will this have for MNEs?

In a nutshell, changes in the geography and composition of global migration have altered the nature of location and firm-specific advantages. A nation’s or firm’s competitive position in terms of labor resources depends not only on the ability to create human capital endowments but also increasingly on the success in competing with other countries and firms to attract and retain such labor (IOM, 2020; Tung, 2016).

### The Implications of Migration for Firms’ Entry Modes and Market Selection Approaches

Our second argument is related to the increasingly transnational character of migrants’ lives and identities, and their concentration in a shrinking number of preferred destination countries (Czaika & de Haas, 2014). Transnational connections among migrants have a considerable economic, sociocultural, and political impact on both the countries that migrants come from and on those in which they live. The economic impacts of migrants are extensive, most significantly in the massive flow of remittances (well over US$700 billion in 2019) that they send to their countries of origin (Cazachevici, Havranek, & Horvath, 2020). Beyond the financial impact, the ongoing links between migrants and their countries of origin also have extensive two-way social and cultural effects (Levitt, 1998) that ultimately may emerge in the politics of the sending and receiving countries, especially on questions of citizenship and homeland issues (Fitzgerald, 2008; Itzigsohn, 2000).

These ongoing processes of economic, sociocultural, and political transformations are enhanced by concentrations of same-nationality migrants in the so-called “global migration magnets”, such as North America, Europe, Australasia, the Gulf states, and the Asian “Tiger” economies (Czaika & de Haas, 2014: 294). Regardless of where they settle, these newcomers often create “migrant spaces” (Saxenian, 2006: 59–60) that, because of their unfamiliarity with the institutions and business operations in their new surroundings, often reflect much of what they left behind. Within such newly created familiarity, they can communicate with others of common backgrounds, learn from their experiences of the new country, or even find their financial footing through business start-ups to serve their ethnic community (Landolt, Autler, & Baires, 1999). The collective social and economic interactions that result can, over time, lead to changes in the destination countries. Migrants are notoriously entrepreneurial (Ram, Jones, & Villares-Varela, 2017), often developing “new” demands among the local population. Their adjustment to their new communities and their adaptation and eventual deployment of what they bring from their countries of origin, along with the institutional and organizational entities they form to serve their own communities, have the potential to reshape elements of their new countries. This makes the location less “foreign” for such migrants, and may in turn positively affect resource investment in these locations by migrants’ country of origin firms (Shukla & Cantwell, 2018).

Few studies in IB have examined the full range of the migration/foreign direct investment (FDI) links (although see Shukla & Cantwell, 2018), emphasizing instead the knowledge mechanisms by which migrants impact FDI activities between their home country and country of destination (Hernandez, 2014). This is important, but there is a need to recognize wider changes in global migration patterns and their implications for the contemporary context in which MNEs operate. As Buckley et al. (2017) have noted, it cannot be assumed that previously identified causal mechanisms and boundary conditions remain unchanged. In theoretical terms, this means that IB research on various cultural and institutional distance measures may need to be reconsidered (Tung & Verbeke, 2010) in the light of changing patterns of global migration, the increasingly transnational character of migrants’ lives, and the potentially decreasing liability of foreignness (Zaheer, 1995).

The interrelated nature of societal contexts, migrants, and host-country nationals, and the way they influence each other in cross-cultural exchanges, also call into question the comprehensiveness of our studies of the causal relationships between international environments and the activities, strategies, and decision-making processes of MNEs. Factors such as the presence of same-nationality migrants and their transnational character may be important for firms’ international success, and in some cases as relevant as factors such as political stability, economic development, quality of formal institutions, and the geographic, cultural, or linguistic proximity typically studied in much more detail in the extant IB literature (Shukla & Cantwell, 2018). Given that sensitivity to comprehensiveness is the hallmark of a good theoretician (Whetten, 1989), there is room here for the expansion of IB research.

We examine what we consider to be three important avenues for future research in this domain: (1) migration and the internationalization development path; (2) how the migration-trade nexus can incorporate insights from transnationalism; and (3) constructing and deconstructing migration and the migration–export relationship.

#### Migration and the internationalization development path

In terms of antecedents or predictors of FDI activities for firms, the foreign operations/migration link may be different for firms from migrant-sending (predominantly emerging market) countries. Gao et al. (2013) studied the impact of human mobility on Chinese FDI and found that Chinese firms that have accumulated capital during their development use FDI as a strategy to obtain knowledge-related assets overseas and thereby increase their competitiveness. They rely on social, business, and familial ties with same-nationality migrants abroad to obtain access to new knowledge networks (Shukla & Cantwell, 2016). Likewise, Hernandez (2014) examined foreign subsidiaries founded in the USA by firms from 27 countries. He found that commonalities between settled migrants and firms in their countries of origin were important factors in foreign firms’ overall internationalization efforts, especially for firms inexperienced in the destination countries and for firms highly dependent on knowledge transfer. The nascent evidence on this topic raises several interesting research questions. Do changes in global migration patterns suggest possible contextual limits to internationalization theories? Does concentration of same-nationality migrants in a destination country aid the emerging market MNEs’ “springboard” approach to international expansion (Luo & Tung, 2007)? What are the implications of shifts in the geography and composition of global migrants for the ownership and location advantages of firms from net emigration countries? Have migratory movements over the last seven decades triggered any shifts in competitive advantages between firms from migrant-sending (primarily emerging market) economies and those from migrant-receiving (predominantly high-income) countries?

Nearly 70% of international migrants live in high-income countries. The two Asian countries, India and China, had the biggest absolute numbers of migrants residing abroad (IOM, 2020). Taking China as an example, Chinese immigration into the USA has grown nearly sevenfold since 1980. Standing at 45 million people in 2018, Chinese represented the third largest group of first-generation immigrants in the USA (Echeverria-Estrada & Batalova, 2020). As consumers, Chinese migrants in the USA stimulate trade by buying products from their country of origin. They are also an important element of a wider process of an increasing attractiveness of home-country products in US markets because of their presence, generating interest in Chinese products among nationals of the destination country (Cai et al., 2021). The more educated and widely dispersed migrants are, the greater their social impact on the local communities (Portes, 2003). Combined, these effects likely impact the internationalization process of Chinese firms.

In summary, MNEs are embedded in social systems that are inevitably influenced by migration (Goldscheider, 1987). The relationship between social systems and migration reflects the varieties of migration types, the complexity of social structures, and the reciprocal ways migration and social structures are interrelated over time. It is one of the contextual features of the environment in which MNEs operate: And we know that context affects the existence, direction, and strength of any theorized effects (Buckley & Lessard, 2005). Further research that includes migration effects may provide a better understanding of the interrelationship between migration and firms’ internationalization development paths.

#### How the migration-trade nexus can incorporate insights from transnationalism

Migrants boost international trade in three different ways. First, they help reduce informal barriers arising from culture, language, or institutional differences. They can help establish business relationships, and they may share important data on sourcing opportunities and foreign sales (Gould, 1994). Second, migrants promote trade if they attach more value to products produced in their home countries (Felbermayr & Toubal, 2012). Third, migrants act as “creators of institutional affinity” and “connectors of institutional environments.” “Over time, they bring about changes in the institutional environment of a location, which makes the location less foreign and more attractive for investing firms” (Shukla & Cantwell, 2018: 835).

With one notable exception (Cai et al., 2021), scholars have not examined time or duration effects on the trade channel as migrants integrate locally. Cai et al. (2021) found that less-integrated migrants have a greater preference for goods from their original home country, while more-integrated migrants influence consumption patterns in their destination country by generating interest in country-of-origin products among locals in their new home. It should be noted that the authors used a proxy for integration, namely the percentage of the migrant population that uses Chinese as the main language of communication. Although language is a relevant metric, it may not be the ideal indicator of time or duration effects on the trade channel. In addition, transnational activities that drive migrants’ integration outcomes are not necessarily transitional. For example, evidence reveals that every additional year a migrant spends in the USA leads to a 3.5% increase in transnational political activities. Furthermore, there are also major differences among different immigrant nationalities. For example, Dominicans and Salvadorans in the USA are more likely to take part in cross-border political activities than Colombians, with both Dominicans and Salvadorans displaying a significantly greater propensity to support sociocultural initiatives linking them to their countries of origin (Portes, 2003). Scholars who study the trade migration nexus should incorporate insights from transnationalism into their theorizing.

There are important implications here for policymakers, especially those who advocate for the conventional assimilation perspective that considers diversity as a threat to social cohesion and therefore demands a high degree of adjustment by migrants (Chand & Tung, 2019; IOM, 2020). This fails to take into consideration that, once migrants have fully assimilated, the pro-trade effects of their immigration are likely to disappear, with potentially negative economic and social consequences. We need more evidence of the impact of time and integration policies on the pro-trade effects of migration.

#### Constructing and deconstructing migration and the migration-export relationship

To date, scholars who have studied the relationship between the co-movement of migrants and cross-border exports, imports, and investment have predominantly relied on an aggregated approach to provide an overall picture of internationalization at the country level (for exceptions, see, e.g., Cai, et al., 2021; Hernandez & Kulchina, 2020). Hernandez and Kulchina (2020) examined how independent foreign firms owned by individual foreigners, and MNE subsidiaries owned by a foreign corporate parent, benefit from immigrants. They found that independent firms rely on the personal connections of their individual managers to access resources from the immigrant community. Subsidiaries of MNEs, on the other hand, use organizational resources of their parent firm, such as brand and reputation, to obtain immigrant community resources. Similarly, Cai et al. (2021) discovered that individually owned and remotely located Chinese firms that have limited external connections or restricted resources benefit more from same-nationality migrations abroad than state-owned firms and firms located in well-connected regions of China. In short, not all firms benefit equally from same-nationality migrants abroad.

Scholars should continue to explore what the deconstruction of migration can tell us about the key underlying processes that connect migration and exporting at the firm level. It would be helpful to have a deeper understanding of issues such as: immigrants’ cultural values and norms, motives for migration, and time since migration; of whether immigrants reside in nations built on historical immigration, such as the USA or Australia, compared with nations with more recent immigrants such as in the Arab Gulf region; of whether they come from countries that used to be a colony of the other; and of the distinct role of migrants as potential customers of exporters as opposed to their potential role as business facilitators. The framing of future studies should use such disaggregated constructs and move away from simply examining aggregated associations between migration and exporting. Few of these distinctions have been addressed in the IB literature, and they therefore offer rich research opportunities.

### Migration and Firms’ Organizational Culture and Approach to Strategy

Our third argument relates to the inherent diversity that now characterizes migrants and the specific concentration of migrant cohorts in so-called “global cities.” Although many migrants, of course, share elements of common identity – especially those elements referring to shared cultural and linguistic backgrounds arising from a common country of origin – it has also been suggested that, for cohorts of contemporary migrants whose social worlds encompass more than one place, identities are negotiated. Referred to as a “multi-local life-world” (Vertovec, 2001), its existence is said to generate a more complex set of influences on the construction of multicultural identities than understood heretofore. Such identities impact how individuals act and position themselves on an ongoing basis within and across their different places of attachment and belonging.

Most contemporary migrants are destined for cities. It is in the urban context that migrants’ human capital is most rewarded (IOM, 2018). For example, longstanding migrant communities (migrants or children of migrants) represent half of Vienna’s population, while over a third of people living in London are migrants, from 200 other countries (OECD, 2018; Pariona, 2018). Multinationals usually establish their (regional) headquarters in cosmopolitan areas that host highly educated and diverse migrant workforces. In some organizations, the number of international migrants exceed the number of local country employees (Zilinskaite & Hajro, 2022), with consequent implications for the culture, activities, strategies, and decision-making processes of the MNEs themselves.

Migrants inevitably introduce numerous differences in culture into their receiving countries. The same individual differences that reflect their various backgrounds are also carried into organizations they join, consequently affecting the internal processes of how these organizations behave and ultimately perform (Jelinek & Wilson, 2005). This internal navigation of cultural differences within teams and organizational units impacts the macro-organizational context, with migrants potentially challenging corporate values and norms and serving as catalysts for organizational change (Hajro, Gibson, & Pudelko, 2017).

The number of enterprises established by migrants is also rapidly increasing (Vandor & Franke, 2016). Taking the USA as an example, 17% of entrepreneurial businesses were launched by immigrants in 1995, increasing to 27% by 2008. In high-growth start-ups backed by venture capital firms, immigrant firms have performed especially well (Kerr, 2019; Kerr & Kerr, 2017). Given that founders have a strong imprinting influence on firms’ values (Schein, 1993), the cultural values and norms of this immigrant entrepreneurial cohort, acquired in the home country, are likely to spill over to the new enterprise. Indeed, new ventures launched by immigrants commonly trade with the immigrant’s home country from their very founding.

These insights contrast with the generally accepted wisdom that national contexts determine how firms are created, managed, and organized (Caprar, Devinney, Kirkman, & Caliguiri, 2015; Porter, 1990). However, they are in line with theories and studies that have reported that attitudes, perceptions, and behaviors can spillover from one context to another (Ilies, Wilson, & Wagner, 2009). These new realities and their implications for IB theorizing need to be given fuller expression in future IB research. They raise several important questions. What insights can be gained from exploring the interface between cities as economic hubs (Goerzen et al., 2013; Sassen, 2018), their relationship with transnational migrant communities, and the effects on MNEs? Is national culture losing traction as a predictor of firm-level tendencies to create, organize, and manage firms’ operations? What are the implications of the rise in the multiplicity of migrants’ countries of origin – a phenomenon conceptualized in social sciences as “superdiversity” – for cross-border activities of MNEs (Vertovec, 2007)?

For example, IB research on cross-border mergers and acquisitions (M&As) has historically assumed headquarters’ employees are representative of their home countries, implying that national culture influences them and the organizations that employ them in a generalizable way, thereby allowing for relatively simple cross-country comparisons. Empirical research into the common understanding that national cultural differences reflect deep differences in values and norms, and thus may cause serious culture clashes in cross-border M&As (Lee, Kim, & Park, 2015), has been far from conclusive (Malhotra & Gaur, 2014; Xie, Reddy, & Liang, 2017). This may be partly because studies have not considered the multidimensional shifts in migration patterns that have resulted in an unprecedented heterogeneity among today’s workforces in MNEs. Migrants’ values, norms, assumptions, attitudes, and beliefs can weaken the influence of societal-level national culture at the headquarters. At the same time, they can serve to increase and enrich the range of viewpoints, routines, and practices available to the MNE, and thus deepen its general cross-cultural knowledge stock.

Overall, therefore, the contemporary social complexity that characterizes today’s MNEs may be a more powerful predictor of their potential cross-border performance gains than cultural distance *per se* and could serve as an interesting avenue for future research.

## Conclusions

Most people – 96.4% of the global population – “do not migrate, despite the economic models based on ‘push’ and ‘pull’ factors of wage and unemployment differentials that predict that they should go” (King, 2012: 5). As Castles, de Haas, & Miller (2014) point out, the number of international migrants has increased only somewhat more rapidly than total global population growth since 1960. What has significantly changed, however, is the geography and composition of global migrants. And it is exactly these changes that have transformed the contemporary context within which MNEs operate, with implications for the activities, strategies, and decision-making processes of firms. In calling attention to these dynamics, we hope to encourage a deeper and more compelling IB narrative on migration.

The increasingly transnational character of migrants’ lives and their concentration in a decreasing number of destination countries have had significant implications for the cross-border activities of firms. IB scholars have begun to respond to these new realities. We have provided an overview of where we currently stand, and have delineated several avenues for research. Identifying current shortcomings in IB research is a first task: “Pushing beyond should be the major goal” (Buckley, 2016: 80).

One requirement for future research will be the need for an awareness of what has been researched on international mobility in other disciplines, and a cross-disciplinary effort to benefit from that understanding in IB, to further contextualize IB, and to infuse other disciplines with a fuller account of the potential role of IB in understanding the impacts of migration. Disciplines with a longer history of studying migration can provide important explanatory power that can inform emerging understandings that are salient to IB scholarship. The perspectives accumulated in sister fields over many years have given migration its own identity and highlighted its unique characteristics. Following the best traditions, with which many IB scholars have a natural affinity, we need to build upon these insights and engage in meaningful cross-disciplinary collaborations if we wish to extend current conversations on global migration and IB and develop novel, bold, and meaningful contributions.

The IB community has, as we have indicated, much to offer to the current cross-disciplinary conversation on this topic. It is not without reason that its perspectives have been asked for (Scott, 2013). It is now time that IB scholars mobilize their efforts, acknowledge the interdependencies among business, migration, and society, and reconsider some of the assumptions underlying their theories with a view to embracing their innate scholarly capacity as both *builders* in generating new perspectives and/or *expanders* in refining existing theories (Colquitt & Zapata-Phelan, 2007). This will allow them to regain their “past strengths” and create new rigorous and relevant aspirational work with potential to stretch into other disciplines (Buckley et al., 2017: 1045). We look forward to learning from these efforts.

## Notes

1. The authors use the US Office of Science and Technology Policy (2014) definition of grand challenges as ‘‘ambitious but achievable objectives that harness science, technology, and innovation to solve important national or global problems and that have the potential to capture the public’s imagination.’’
2. To this end, we reviewed articles published in leading migration-specific academic journals (e.g., *International Migration Review*, *Journal of Ethnic and Migration Studies*), as well as widely featured books on migration (e.g., *The Age of Migration: International Population Movements in the Modern World*). Building on this body of evidence, we then moved on to review work within the IB field on migration. We identified and reviewed 41 articles published in the *Journal of International Business Studies, International Journal of Business Policy, Journal of World Business, International Business Review, International Journal of Management Reviews,* and *Management International Review*. We also analyzed selected reports from the World Bank, International Organization for Migration (IOM), International Labor Organization (ILO), Institute for Human Rights and Business, and the Responsible Business Alliance. In addition, we drew upon overview and review articles by Barnard, Deeds, Mudambi, and Vaaler (2019), Hajro et al. (2021), and Hajro, Stahl, Clegg, and Lazarova (2019) on migration in management and IB journals. The theoretical inferences and integrative recommendations we advance here are grounded in the evidence this search revealed. It also serves as the wellspring for the identification of the key future research opportunities that we believe, viewed through an IB lens, could deepen our understanding of this important domain area.
